# Loss of p300/CBP-associated factor aggravates cardiac remodeling via regulation of CAMKK2 acetylation

**DOI:** 10.1038/s12276-026-01698-z

**Published:** 2026-04-20

**Authors:** Yongwoon Lim, Anna Jeong, Duk-Hwa Kwon, Yun-Gyeong Lee, Yeong-Un Lee, Hye Jung Cho, Hae Jin Kee, Somy Yoon, Ho-Geun Yoon, Yugyeong Kim, Sang Beom Seo, Kwang-Il Nam, Gwang Hyeon Eom, Youngkeun Ahn, Jeongsik Yong, Young-Kook Kim, Hyun Kook

**Affiliations:** 1 Department of Pharmacology, Chonnam National University Medical School, Hwasun, Republic of Korea; 2 Medical Research Center for Innovative Control of Cardiovascular Remodeling Diseases, Hwasun, Republic of Korea; 3 Department of Anatomy, Chonnam National University Medical School, Hwasun, Republic of Korea; 4 Department of Cardiology, Heart Research Center, Chonnam National University Hospital, Gwangju, Republic of Korea; 5 College of Pharmacy, Chonnam National University, Gwangju, Republic of Korea; 6 Department of Biochemistry and Molecular Biology, Graduate School of Medical Science, Brain Korea 21 Project, Yonsei University College of Medicine, Seoul, Republic of Korea; 7 Department of Life Science, College of Natural Science, Chung-Ang University, Seoul, Republic of Korea; 8 Department of Cardiology, Chonnam national University Hospital, Gwangju, Republic of Korea; 9 Department of Biochemistry, Molecular Biology and Biophysics, University of Minnesota Twin Cities, Minneapolis, MN USA; 10 Department of Biochemistry, Chonnam National University Medical School, Hwasun, Republic of Korea; 11 Present Address: R&D Center PODO Therapeutics Co., Seongnam-si, Republic of Korea

**Keywords:** Acetylation, Mechanisms of disease

## Abstract

Here we aim to elucidate the role of the p300/CBP-associated factor (PCAF) in pathological cardiac remodeling. Specifically, we explore how PCAF-mediated acetylation of calcium/calmodulin-dependent protein kinase kinase 2 (CAMKK2) influences AMPK signaling, thereby regulating cardiac hypertrophy and dysfunction under pathological stress. A genetically engineered PCAF-knockout (KO) mouse model was generated using the CRISPR–Cas9 system to evaluate the effect of PCAF deficiency on cardiac remodeling induced by isoproterenol infusion and transverse aortic constriction (TAC). PCAF deficiency significantly aggravated cardiac enlargement with features of eccentric hypertrophy, as demonstrated by histological analysis and echocardiography. To determine these phenotypes were cardiomyocyte specific, we generated a cardiomyocyte-specific conditional KO model, which also showed a dilated cardiomyopathy-like phenotype similar to that of the global-KO mice. Transcriptomic analysis of TAC-operated hearts from wild-type and KO mice revealed enrichment of pathways related to mitochondrial function and energy homeostasis. Mechanistically, PCAF directly acetylated CAMKK2, promoting its activation and the subsequent phosphorylation of AMP-activated protein kinase α (AMPKα) at Thr172, a critical step in maintaining metabolic balance under stresses. These signaling alterations were also observed in the hearts of PCAF-KO hearts subjected to isoproterenol administration or TAC. Pharmacological activation of PCAF with SPV106 effectively attenuated TAC-induced cardiac remodeling, preserving cardiac structure and function. Collectively, these findings identify PCAF as a pivotal regulator of pathological cardiac remodeling through modulation of the CAMKK2–AMPK signaling axis. Loss of PCAF exacerbates stress-induced cardiac hypertrophy and dysfunction, highlighting its potential as a therapeutic target to preserve cardiac function and counteract stress-induced remodeling.

## PCAF Deficiency Exacerbates Cardiac Remodeling Under Stress

Heart failure is a condition where the heart can't pump blood effectively, often due to changes in heart structure. Researchers have found that a protein called PCAF might play a role in heart health. The researchers created mice without the PCAF gene and observed how their hearts responded to stress. They found that these mice had worse heart problems compared to normal mice. The study showed that PCAF helps regulate another protein, CAMKK2, which is important for heart energy balance. The results suggest that PCAF protects the heart from damage by maintaining energy balance through CAMKK2. Researchers concluded that enhancing PCAF activity could be a potential treatment for heart disease. In the future, therapies targeting PCAF might help prevent or treat heart failure by improving heart function and structure.

## Introduction

Heart failure refers to the loss of myocardial function, accompanied by myocardial remodeling, such as changes in cardiac shape and fibrosis^1^. The remodeling process is often initiated as a compensatory response to pressure or volume overload^2^. The contractile function of the heart can be evaluated by echocardiography, especially through the ejection fraction (EF). In many cases of early cardiac hypertrophy, EF is relatively preserved, indicating inadequate filling despite a (sub)normal EF. By contrast, when the heart becomes dilated and its contractile function is impaired, it is termed heart failure with reduced EF (HFrEF). In both types, cardiac remodeling plays a crucial role, and its modulation remains a key therapeutic goal.

Impairment of Ca^2+^ homeostasis is regarded as a major contributor to cardiac hypertrophy and heart failure by affecting Ca^2+^ binding proteins. The leading causes of death in patients with heart failure are closely associated with disrupted Ca^2+^ homeostasis^3^. Altered intracellular Ca^2+^ concentrations activate the multifunctional calcium/calmodulin-dependent protein kinase (CaMK) family, as well as CaMK kinases (CaMKKs). In particular, CAMKKs initiate the calcium-signaling cascade by phosphorylating and activating CAMKI and CAMKIV^4–6^. Two CAMKK isoforms (CAMKK1 and CAMKK2, also known as CAMKKα and CAMKKβ) have been identified in mammals, and CAMKK2 has been well established as an upstream kinase of adenosine monophosphate (AMP)-activated protein kinase (AMPK)^7,8^, which is reported to prevent cardiac hypertrophy by regulating energy homeostasis^9–11^. Recent research revealed that cardiac-specific inhibition of CAMKK2 activity accelerated transverse aortic constriction (TAC)-induced cardiac hypertrophy and heart failure^12^. Another study demonstrated that pharmacological activation of CAMKK2 alleviated diabetic cardiomyopathy^13^. However, the upstream regulatory mechanism of CAMKK2 in cardiac hypertrophy and dysfunction is largely unknown.

Protein acetylation is a reversible post-translational modification of proteins of lysine residues by acetyltransferases, altering protein properties. It is involved in diverse processes, including transcriptional regulation, stability, metabolism and stress response^14^. Aberrant acetylation is associated with the development of cardiac diseases, such as cardiac hypertrophy, cardiac fibrosis and heart failure. Previously, we reported that p300/CBP-associated factor (PCAF) induces activation of histone deacetylase 2 (HDAC2), a prohypertrophic molecule, via acetylation under hypertrophic conditions^15^. PCAF is a member of general control non-derepressible 5 (GCN5)-related N-acetyltransferase, playing multiple roles in biological processes, including cell cycle, cell differentiation and cell metabolism. A recent study showed that genetic variation in PCAF is linked to reduced vascular morbidity and mortality, including coronary heart diseases^16^. For instance, we previously demonstrated that PCAF directly acetylates SMAD2, a profibrotic mediator of TGF-β, thereby regulating the fibrotic signaling pathway in primary human cardiac fibroblasts^17^.

Notably, PCAF has been recently implicated in dystrophic and ischemic heart disease^18,19^. However, findings regarding its role in cardiac hypertrophy and remodeling remain inconsistent. Some studies have reported that PCAF induces cardiac hypertrophy via hyperacetylation of histone H3K9ac in vitro^20^. Likewise, we previously found that PCAF can induce cardiac hypertrophy in vitro by acetylating and activating HDAC2^15^. By contrast, it was reported that PCAF- and P300-mediated acetylation of AKT is important to prevent cardiac hypertrophy in vitro^21^. This discrepancy may reflect differences in cell models and the lack of in vivo genetic evidence. Hence, further research using genetically engineered mouse models is needed to clarify the pathological roles of PCAF in cardiac remodeling. Here, we investigated the function of PCAF in vivo using global PCAF-knockout (KO) and cardiomyocyte-specific conditional KO (CKO) mice.

## Materials and methods

### Antibodies and reagents

The antibodies used were as follows: PCAF (#3378S), AMPK (#2532), phospho-AMPKα (pAMPKα) (Thr172) (#2531), liver kinase B1 (LKB1) (#3047), phospho-LKB1 (pLKB1) (Ser428) (#3482), CAMKK2 (#16810), phospho-Acetyl-CoA carboxylase (ACC) (Ser79) (#3661), ACC (#3676) and acetylated-lysine (AcK) (#9814), from Cell Signaling Technology; Flag (#F1804) and HA (#H9658), from Thermo Fisher Scientific; acetyl-lysine (#ab21623), from Abcam; GAPDH (#sc-365062), β-actin (#sc-47778), normal mouse IgG (#sc-2025) and normal rabbit IgG (#sc-2027), from Santa Cruz Biotechnology; horseradish peroxidase (HRP)-conjugated secondary antibody against mouse IgG or rabbit IgG was from Cell Signaling Technology.

Hyaluronidase was from Worthington Biochemical. Collagenase type B was from Hoffmann-La Roche. Isoproterenol (ISP) hydrochloride, bovine serum albumin, taurine, 2,2,2-tribromoethanol, 2-metyl-2-butanol and 2,3-butanedione monoxime were purchased from Sigma. SPV106 was purchased from Merck Millipore.

### Expression of PCAF in human failing heart

The expression of PCAF in human dilated cardiomyopathy (DCM) was examined with public gene expression data from the Gene Expression Omnibus (GEO) database (GDS2205), including subendocardial left ventricular samples from seven patients with DCM and five nonfailing hearts analyzed on the Affymetrix Human Genome U133A Array platform. Differentially expressed genes were identified using a two-class unpaired significance analysis of microarrays, with significance defined as false discovery rate <0.05 and fold change ≥1.2. The Kat2b gene expression was then examined in this dataset.

### Cardiac hypertrophy in vivo model

All animal experiments were approved by the Institutional Review Board of Chonnam National University Medical School Research Institutional Animal Care and Use Committee (CNU IACUC-H-2024-3) and performed according to the Guide for the Care and Use of Laboratory Animals published by the US National Institutes of Health. Male C57BL/6J mice were purchased from Samtako BIO KOREA. Eight-week-old C57BL/6J male mice were infused with 30 mg/kg/day ISP in phosphate-buffered saline (PBS) or the equivalent volume of PBS by Alzet osmotic pump (DURECT Corporation) for 6 days. TAC surgery and echocardiography are described in the Supplementary Methods. 2,2,2-Tribromoethanol (Avertin, #T48402, Sigma) was used for echocardiography, because it is known to have fewer side effects on cardiovascular and hemodynamic stability^22–25^.

Cardiac fibrosis was induced by continuous subcutaneous infusion of ISP (30 mg/kg/day) for 6 days using an osmotic pump, as previously reported^26,27^. The pumps were implanted in the dorsal region of mice and maintained for 6 days. Age-matched control mice were administered an equivalent volume of PBS. Following completion of the infusion period, the animals were euthanized, and heart tissues were collected for subsequent analyses.

After 3 weeks of TAC surgery, wild-type (WT) mice were intraperitoneally injected with the PCAF histone acetylase activator SPV106 (20 mg/kg) every 2 days for 2 weeks. Control animals were injected with saline solution and an equivalent amount of solvent (dimethyl sulfoxide). The concentration of SPV106 used in this study was determined on the basis of the report by Colussi et al.^18^, which demonstrated that administration of SPV106 (10–40 mg/kg) in mice effectively increased cardiac protein acetylation, including acetylated tubulin, without apparent toxicity. Recently, it was reported that SPV106 inhibited calcification and preserved valvular motion and cardiac function in both a vitamin-D-induced aortic valve calcification model and an ex vivo valvular calcification model^28^. Accordingly, this dosage range was referenced, and the optimal concentration for our cardiac fibrosis experiments was established based on the in vivo efficacy reported in previous studies on the heart and cardiovascular system.

### Generation of the PCAF KO

The PCAF KO mouse was generated using the CRISPR–Cas9 system (Toolgen). In brief, single guide RNAs (sgRNAs) were designed to target the second exon of *Pcaf*. The sgRNAs were introduced into the fertilized embryos, along with Cas9 mRNA. The deletion in *Pcaf* was confirmed by Sanger sequencing, and the existence of PCAF protein in various tissues was further confirmed by western blot (WB). Tail genomic DNA was used for genotyping. For each experiment, PCAF-KO mice beyond the 6th generation were used.

### Generation of cardiomyocyte-specific PCAF-KO mice

*PCAF*^*flox/flox*^ mice were generated by Cyagen Biosciences and *Myh6-Cre* (*αMyHC-Cre*) mice were obtained from Dr. Ji-One Kang (Kyung Hee University). To create cardiomyocyte-specific PCAF CKO mice (*Myh6-Cre;PCAF*^*flox/flox*^), we crossed *PCAF*^*flox/flox*^ mice with *Myh6-Cre* mice.

### Isolation of mouse cardiac fibroblasts and myocytes and cell cultures

A Langendorff perfusion was performed to isolate myocytes from hearts as previously described^29^. A detailed description of the method is provided in the Supplementary Methods.

### Histology, immunohistochemistry and TEM analysis

Histological analysis was carried out as previously described^27^. A detailed description of the method is provided in the Supplementary Methods.

For transmission electron microscopy (TEM) analysis, mouse hearts were collected and trimmed, and atria were removed. The left ventricles were fixed in 4% paraformaldehyde, then further fixed in 2.5% glutaraldehyde and 1% paraformaldehyde. Subsequently, fixed samples were postfixed in osmium tetroxide, then embedded in resin, followed by sectioning at 90 nm. TEM images were obtained by a JEOL JEM-2100F field emission transmission electron microscope (JEOL).

### Subcellular fractionation

Subcellular fractionation was performed as described by Dimauro et al.^30^. Hearts were minced and pelleted by simple centrifugation. Samples were homogenized in STM buffer (250 mM sucrose, 50 mM Tris–HCl pH 7.4 and 5 mM MgCl_2_, with protease/phosphatase inhibitors (PIs)). The homogenate was centrifuged, yielding the nuclear fraction and cytosolic/mitochondrial fractions. For further purity, the nuclear fraction was resuspended in NET buffer (20 mM HEPES pH 7.9, 1.5 mM MgCl_2_, 0.5 M NaCl, 0.2 mM EDTA, 20% glycerol, 1% Triton-X-100 and PIs) and lysed to isolate the nuclear fraction. Cytosolic/mitochondrial fractions were centrifuged, and the supernatant was collected for cytosolic fraction. To obtain the cytosolic fraction, the supernatant was precipitated with acetone, followed by centrifugation. For the mitochondrial fraction, pellets from cytosolic/mitochondrial fractions were resuspended in the STM buffer (250 mM sucrose, 50 mM Tris–HCl pH 7.4, 5 mM MgCl_2_ and PIs), followed by centrifugation. Then, pellets were resuspended in SOL buffer (50 mM Tris–HCl pH 6.8, 1 mM EDTA, 0.5% Triton-X-100 and PIs) and sonicated to obtain the mitochondrial fraction.

### RNA analysis, WB and IP

Protocols for RNA extraction and quantitative real-time polymerase chain reaction (qRT–PCR), WB and immunoprecipitation (IP) are described in the Supplementary Materials.

### In vitro acetylation assay

Recombinant GST-CAMKK2 and GST-PCAF (HAT domain, 1052-2499 bp) were expressed in *Escherichia coli* BL21(DE3), followed by purification using Glutathione Sepharose 4B (#17075601, Cytiva). The in vitro acetylation assay was performed at 30°C for 3 h in 40 µl containing 250 mM Tris–HCl (pH 8.0), 2.5 mM EDTA, 25 mM dithiothreitol, 50% glycerol and 100 µM acetyl-CoA (#A2056, Sigma). Proteins were separated using sodium dodecyl sulfate–polyacrylamide gel electrophoresis, and transferred to a nitrocellulose membrane. The membrane was incubated at 4 °C with anti-AcK antibody (#sc-81623, Santa). Then, the membrane further was incubated with HRP-conjugated secondary antibody and detected.

### Statistics

Statistical analysis was performed using PASW Statistics 26 (SPSS, IBM). Data are presented as means ± standard error of the mean (s.e.m.). To compare two independent groups, we used a two-tailed unpaired Student’s *t*-test or nonparametric Mann–Whitney *U* test after checking for a normal distribution. To compare more than two groups, we used one-way analysis of variance (ANOVA) or two-way ANOVA with post-hoc tests according to the levels of independent variables. When the interaction between independent variables was significant, stratification was performed for pairwise comparison. The assumption of equal variance was confirmed using Levene’s test. For post-hoc tests, we performed Tukey’s honestly significant difference (HSD) for multiple comparisons with equal variance, whereas the Dunnett’s T3 test was used for unequal variance. Statistical significance was considered when the *P* value was <0.05. In figures, asterisks indicate the level of statistical significance: ns, not significant, *P ≤ 0.05, **P ≤ 0.01, ***P ≤ 0.001 and ****P ≤ 0.0001.

## Results

### PCAF is downregulated in cardiac hypertrophy

**Fig. 1:**
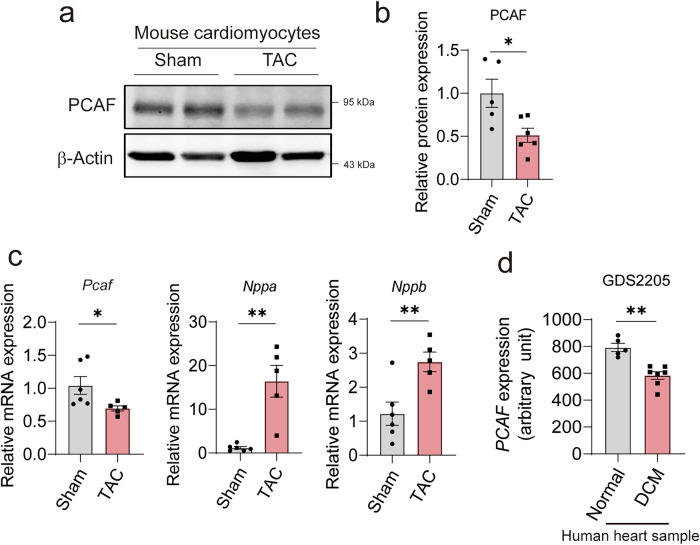
Suppression of PCAF in a cardiac hypertrophy in vivo model. Mice were subjected to TAC surgery for 8 weeks. **a** Representative WB images of cardiomyocytes isolated from mouse hearts. **b** Quantification of PCAF protein expression in isolated cardiomyocytes (*n* = 5–6 per group). **c** qRT–PCR analysis of mRNA levels of PCAF and hypertrophic markers in isolated cardiomyocytes (*n* = 5–6 per group). **d** Relative expression of PCAF in a clinical dataset consisting of ventricular samples from healthy donors and patients with DCM (*n* = 5–7 per group). *P* values were determined by Student’s *t*-test. All data are presented as mean ± s.e.m.

In our previous study, we showed that PCAF acetylates HDAC2, thereby inducing cardiac hypertrophy in vitro^15^. Thus, we first investigated whether PCAF is related to cardiac hypertrophy in vivo, using the TAC-induced cardiac hypertrophy in vivo model^31^. WT mice subjected to TAC surgery showed significant increases in the ratio of heart weight to tibia length (HW/TL) and the ratio of lung weight to TL (LW/TL), which is indicative of heart failure^32^ (Supplementary Fig. 1a,b). Echocardiography showed that TAC-operated mice exhibited cardiac dysfunction, indicated by decreased fractional shortening (FS) and EF values (Supplementary Fig. 1c). To determine how PCAF is involved in cardiac hypertrophy, the expression level of PCAF was confirmed in isolated cardiomyocytes from the hearts after TAC. The protein level of PCAF was significantly downregulated in the TAC-treated hearts (Fig. 1a,b). Consistently, qRT–PCR demonstrated that the mRNA level of PCAF was significantly repressed by TAC, accompanied by significant upregulations of hypertrophic marker genes, such as *Nppa* and *Nppb* (Fig. 1c). Next, we examined the expression of PCAF in human DCM, using public gene expression data from the GEO database (GDS2205). We found that PCAF expression was significantly decreased in the ventricles from patients with DCM, compared with hearts from healthy donors (Fig. 1d). Collectively, these data indicate that PCAF is closely related to cardiac hypertrophy.

### Loss of PCAF exacerbates ISP-induced cardiac hypertrophy

To determine the role of PCAF in cardiac hypertrophy in vivo, we created PCAF-KO mice using the CRISPR–Cas9 system. PCAF-KO mice were generated by deleting 50 bp in the second exonic sequence on the *Pcaf* locus, leading to a frameshift mutation (Supplementary Fig. 2a). Genotype PCR of mouse tail genomic DNA showed a distinct amplicon size for the *Pcaf* gene (463 bp), compared with WT (513 bp) (Supplementary Fig. 2b). We further confirmed whether KO mice express PCAF in various organs. PCAF expression was not observed in the heart and skeletal muscle (Supplementary Fig. 2c). In addition, PCAF expression was not detected in various organs, as shown in Supplementary Fig. 2d.

**Fig. 2:**
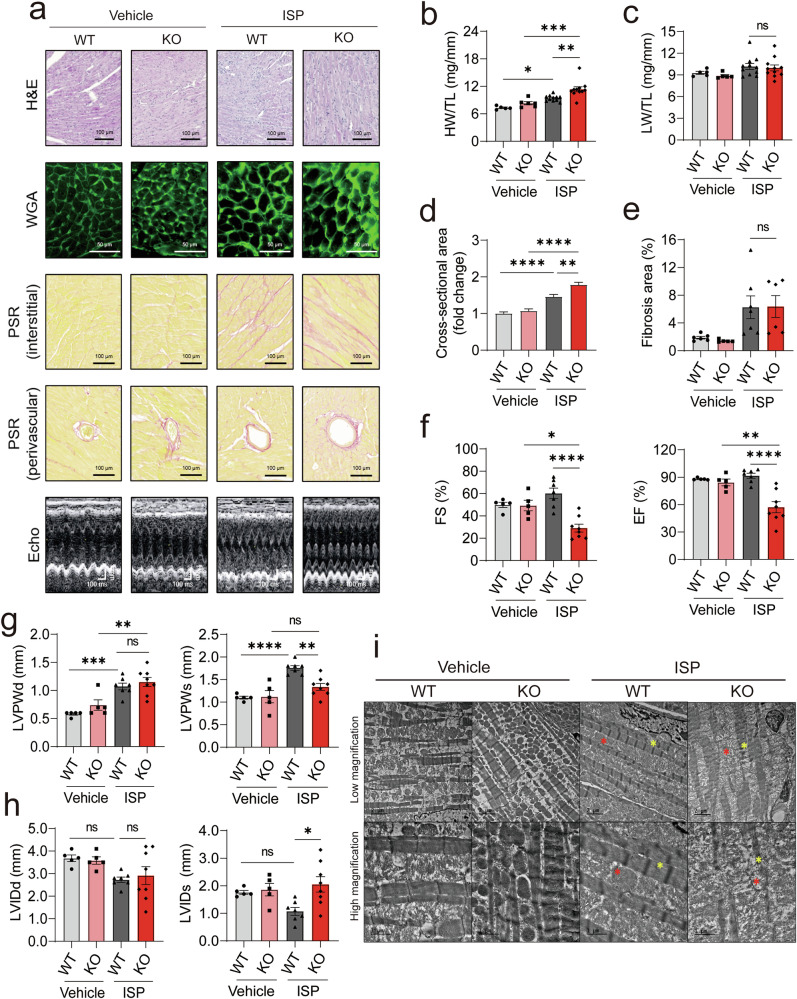
Global deletion of PCAF aggravates ISP-induced cardiac hypertrophy and dysfunction. **a** Representative histological images (hematoxylin and eosin (H&E), wheat germ agglutinin (WGA) and PSR staining) and echocardiography results. **b**, **c** HW/TL (**b**) and LW/TL (**c**) ratios after 6 days of administration with vehicle or ISP (*n* = 5–11 per group). **d** Cross-sectional area of cardiomyocytes analyzed by WGA staining (*n* = 63–100 per group). **e** Fibrosis area (*n* = 6–7 per group). **f**–**h** Echocardiography parameters (*n* = 5–8 per group). Fractionoal shortening (FS) and ejection fraction (EF) (**f**), left ventricular posterior wall thickness at diastole (LVPWd) and systole (LVPWs) (**g**), left ventricular internal diameter at diastole (LVIDd) and systole (LVIDs) (**h**). *P* values were determined using Tukey’s HSD test following one-way ANOVA. All data are presented as mean ± s.e.m. **i** Representative TEM images of hearts after vehicle or ISP treatment. PCAF-KO mice exhibited disrupted sarcomeres and mitochondria under ISP infusion. The yellow asterisk indicates sarcomeres, and the red asterisk indicates mitochondria.

Next, we investigated the effect of PCAF on the development of cardiac hypertrophy. Our previous study demonstrated that PCAF induces cardiac hypertrophy by acetylating HDAC2 in vitro^15^. Therefore, we hypothesized that PCAF-KO mice would exhibit blunted cardiac hypertrophy after ISP treatment. To test this, PCAF-KO mice were implanted with osmotic pumps containing ISP for 6 days to induce cardiac hypertrophy in vivo. Chronic infusion of ISP induced cardiac hypertrophy in WT mice (Fig. 2a and Supplementary Fig. 3), as indicated by increases in the HW/TL ratio and cross-sectional area of cardiomyocytes, but it did not affect the LW/TL ratio, which suggests that overall pulmonary congestion was not developed by ISP. However, contrary to our expectations, these increases were enhanced in PCAF-deficient mice, as shown in Fig. 2a–d. Meanwhile, differences in interstitial and perivascular fibrosis were not observed between WT and KO mice after ISP treatment (see third versus fourth bars in Fig. 2e). To further assess whether PCAF deficiency affects cardiac function, echocardiography was performed. It has been well established that a short-term administration of ISP increases wall thickness with preserved contractile function, leading to an increase in EF and FS values^33,34^. Notably, we found that, in PCAF-KO mice, ISP substantially reduced EF and FS values, suggesting acute cardiac dysfunction after 6 days of ISP treatment compared with WT mice (Fig. 2f). In particular, in WT mice, ISP administration increased the thickness of the left ventricular posterior wall (LVPW) and decreased the left ventricular internal diameter at end-diastole (LVIDd) and at end-systole (LVIDs), suggesting concentric hypertrophy (see first versus third bars in Fig. 2g, left). In PCAF-KO mice, ISP increased diastolic LVPW (LVPWd, second versus fourth bars) to the same extent as it did in WT mice (see first versus third bars in Fig. 2g, left), which suggests that genetic ablation of PCAF did not affect the ISP-induced increase in end-diastolic wall thickness. ISP also increased the systolic LVPW (LVPWs) in WT mice (see first versus third bars in Fig. 2g, right). Interestingly, however, in PCAF-KO mice, ISP failed to increase LVPWs (see second versus fourth bars in Fig. 2g, right), which suggests thinning of the wall.

Although it was not significant, in WT mice, ISP reduced diastolic LVID (LVIDd), representing an increase in wall thickness (see first versus third bars in Fig. 2h, left). However, PCAF KO did not affect this ISP-induced reduction in LVIDd (second versus fourth bars). In WT mice, ISP also reduced systolic LVID (LVIDs; see first versus third bars in Fig. 2h, right). However, ISP failed to reduce LVIDs in PCAF-KO mice (second versus fourth bars). Because ISP-administered WT mice showed an increase in LVPWs and a decrease in LVIDs, the failure of PCAF-KO mice to exhibit an increase in LVPWs or a reduction in LVIDs suggests an impairment of contractile function. TEM analysis further showed that the structure of the sarcomere and mitochondria was disorganized in PCAF-KO mice after ISP treatment (Fig. 2i). Taken together, under ISP treatment, the ablation of PCAF induces cardiac enlargement, which is accompanied by systolic dysfunction.

### Deficiency of PCAF aggravates TAC-induced cardiac hypertrophy and dysfunction

**Fig. 3:**
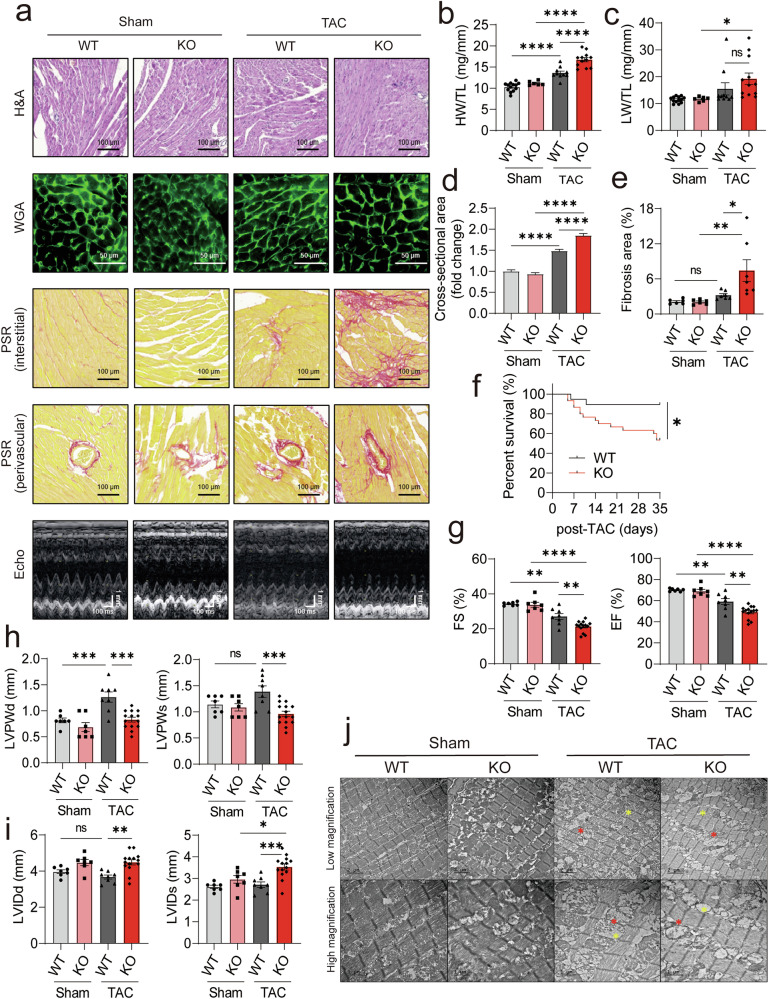
Deficiency of PCAF in mice promotes TAC-induced cardiac hypertrophy and dysfunction. WT or KO mice were subjected to TAC for 4 weeks. **a** Representative histological images (H&E, WGA and PSR staining) and echocardiography results. **b**, **c** Quantification of HW/TL (**b**) and LW/TL (**c**) ratios (*n* = 6–13 per group). **d** Cross-sectional area of cardiomyocytes analyzed by WGA staining (*n* = 89–195 per group). **e** Fibrosis area (*n* = 6–7 per group). **f** Kaplan–Meier survival curves of mice up to 35 days post-TAC (*n* = 19–30 per group). log-rank (Mantel–Cox) tests was used to determine *P* values. For other panels, *P* values were determined using Tukey’s HSD test following one-way ANOVA. All data are presented as mean ± s.e.m. **g**–**i** Echocardiographic parameters (*n* = 7–14 per group). Fractionoal shortening (FS) and ejection fraction (EF) (**g**), left ventricular posterior wall thickness at diastole (LVPWd) and systole (LVPWs) (**h**), left ventricular internal diameter at diastole (LVIDd) and systole (LVIDs) (**i**). **j** Representative TEM images of hearts after sham or TAC surgery. PCAF-KO mice exhibited disrupted sarcomere organization and abnormal mitochondrial morphology after TAC. The yellow asterisk indicates sarcomeres, and the red asterisk indicates mitochondria.

We further validated the function of PCAF in cardiac hypertrophy and function by performing TAC surgery. The TAC operation significantly induced increases in HW/TL and LW/TL ratios in WT mice (Supplementary Fig. 4). PCAF-KO mice showed aggravated TAC-induced cardiac hypertrophy, as determined by increased HW/TL and cross-sectional area of cardiomyocytes, and enhanced pulmonary congestion as indicated by increased LW/TL, a sign of heart failure (Fig. 3a–d). TAC-induced interstitial and perivascular fibrosis were exacerbated by deficiency of PCAF in the mouse (Fig. 3e). After TAC surgery, mortality rates in PCAF-KO mice were significantly higher than in WT (KO 47% versus WT 10% after 4 weeks of TAC) (Fig. 3f). In WT mice, echocardiography revealed that TAC induced a significant reduction in cardiac function, as indicated by FS (see first versus third bars in Fig. 3g, left) and EF values (Fig. 3g, right). Compared with WT (see first versus third bars in Fig. 3g, left and right), TAC-induced decreases in FS and EF were markedly aggravated in KO mice (third versus fourth bars). PCAF-KO mice exhibited more dilated left ventricles than WT mice, as indicated by reduced LVPW and increased LVID, consistent with eccentric hypertrophy. By contrast, WT mice subjected to TAC surgery showed thickening of the cardiac wall, indicative of concentric hypertrophy (Fig. 3h,i). Notably, in contrast to ISP, where changes in LVPW and LVID were observed only during systole (Fig. 2h,i), TAC induced alterations in both diastole (Fig. 3h,i, left) and systole (Fig. 3h,i, right). Failure of thickening of the LVPW indicates an inadequate response to TAC, which may result in ventricular dilation. This ventricular dilation strongly suggests the development of DCM. TEM analysis showed severe damage to mitochondria in PCAF-KO mice after 4 weeks of TAC surgery (Fig. 3j). This result suggests that loss of PCAF may induce both disruption of mitochondrial function and impairment of energy homeostasis. Collectively, these data suggest that PCAF-KO mice are vulnerable to conditions of pathological cardiac hypertrophy and that TAC causes more prominent pathological features, including cardiac hypertrophy and fibrosis.

### Cardiomyocyte-specific deletion of PCAF aggravates TAC-induced DCM and cardiac dysfunction

TAC not only induced cardiac hypertrophy but also provoked a rapid transition to a DCM phenotype, characterized by marked thinning of the LVPW (Fig. 3h,i) and significantly reduced survival, indicating severe heart failure (Fig. 3f). However, cardiac stress such as TAC affects not only cardiomyocytes but also cardiac fibroblasts, another critical cell type in the heart, whose activation profoundly contributes to cardiac remodeling^35,36^. As shown in Fig. 3a (picrosirius red (PSR) staining) and Fig. 3e, the abrupt and extensive myocardial fibrosis suggests that PCAF may also directly influence cardiac fibroblast activation. Cardiac fibrosis typically arises from the aberrant activation of fibroblasts in response to cardiac stress^37–39^, which can occur either through direct effects on fibroblasts^40–42^ or indirectly as a reactive or compensatory response to cardiomyocyte injury^37,43,44^. Therefore, distinguishing whether PCAF acts primarily in cardiomyocytes or exerts a direct profibrotic influence on fibroblasts is of key importance.

Our previous study using cardiac fibroblasts demonstrated that PCAF acetylates and activates SMAD4, thereby promoting cardiac fibrosis^17^. Paradoxically, in the current study, loss of PCAF further aggravated myocardial fibrosis, which appears contradictory to the previous observation. To clarify this discrepancy, we generated cardiomyocyte-specific PCAF-KO mice (*Pcaf*^*flox/flox*^ crossed with *Myh6-Cre* mice, hereafter referred to as CKO; Supplementary Fig. 5) to investigate the cardiomyocyte-specific role of PCAF in TAC-induced stress responses. The targeting strategy and generation process for *Pcaf*^*flox/flox*^ mice are shown in Supplementary Fig. 5a,b. After isolating hearts from CKO mice using the Langendorff perfusion method, we separated cardiomyocytes and noncardiomyocytes (predominantly cardiac fibroblasts) and examined PCAF expression in each population. As shown in Supplementary Fig. 5c, PCAF expression was specifically ablated in cardiomyocytes of CKO hearts.

**Fig. 4:**
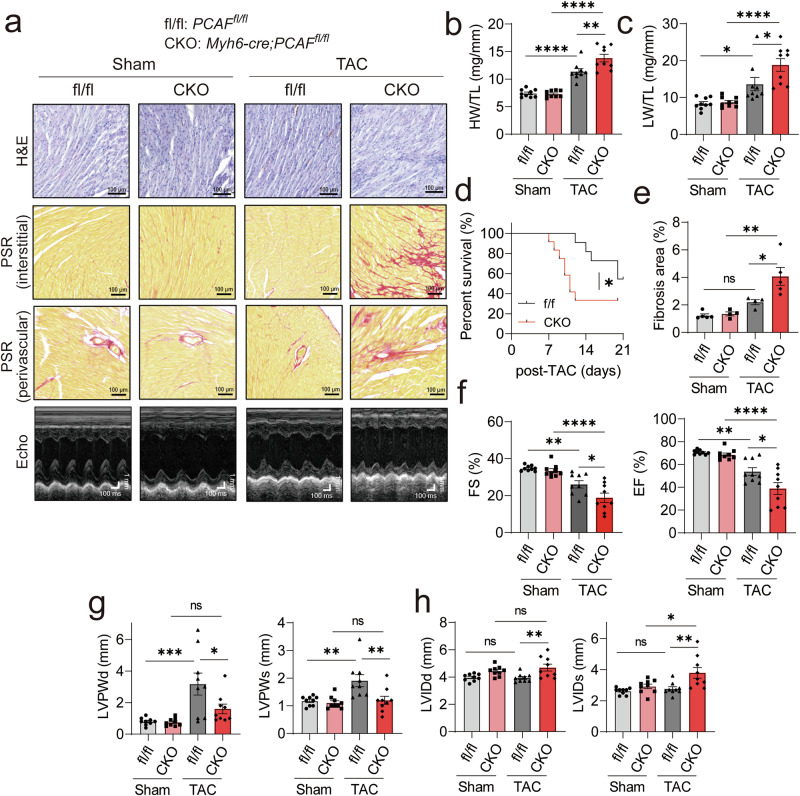
Cardiomyocyte-specific PCAF deficiency (CKO) exacerbates TAC-induced cardiac remodeling. Cardiomyocyte-specific CKO (*Myh6-cre;PCAF*^*fl/fl*^) mice and their WT littermate control (fl/fl; *PCAF*^*fl/fl*^) were subjected to TAC for 3 weeks. **a** Representative histological images (H&E and PSR staining) and echocardiography results. **b**, **c** Quantification of HW/TL (**b**) and LW/TL (**c**) ratios (*n* = 8–9 per group). **d** Kaplan–Meier curves of mice up to 21 days post-TAC (*n* = 11–12 per group). log-rank (Mantel–Cox) tests was used to determine *P* values. For other panels, *P* values were determined using Tukey’s HSD test following one-way ANOVA. All data are presented as mean ± s.e.m. **e** Fibrosis area (*n* = 4–5 per group). **f**–**h** Echocardiographic parameters (*n* = 9 per group).Fractionoal shortening (FS) and ejection fraction (EF) (**f**), left ventricular posterior wall thickness at diastole (LVPWd) and systole (LVPWs) (**g**), left ventricular internal diameter at diastole (LVIDd) and systole (LVIDs) (**h**).

As shown in Fig. 4a–c, both cardiac hypertrophy and pulmonary congestion were further exacerbated in CKO mice following TAC. The gross heart appearance is shown in Supplementary Fig. 6. Compared with control littermate mice (*Pcaf*^*flox/flox*^; hereafter referred to as fl/fl), the survival rate after TAC was markedly reduced in CKO (Fig. 4d). TAC-induced interstitial and perivascular fibrosis was further aggravated in CKO mice (Fig. 4e). Echocardiography revealed that the TAC-induced declines in FS (Fig. 4f, left) and EF (Fig. 4f, right) were significantly more pronounced in CKO mice. Notably, in CKO hearts, TAC failed to elicit thickening of the LVPW, leading to a DCM with eccentric hypertrophy (Fig. 4g), with a concomitant increase in left ventricular internal dimensions (Fig. 4h). These results indicate that the cardiac phenotype observed in CKO mice phenocopies that of global PCAF-KO hearts, suggesting that the detrimental response to TAC arises primarily from the loss of PCAF function within cardiomyocytes rather than from secondary effects on cardiac fibroblasts.

### PCAF-KO mice exhibit disrupted AMPK signaling pathway

**Fig. 5:**
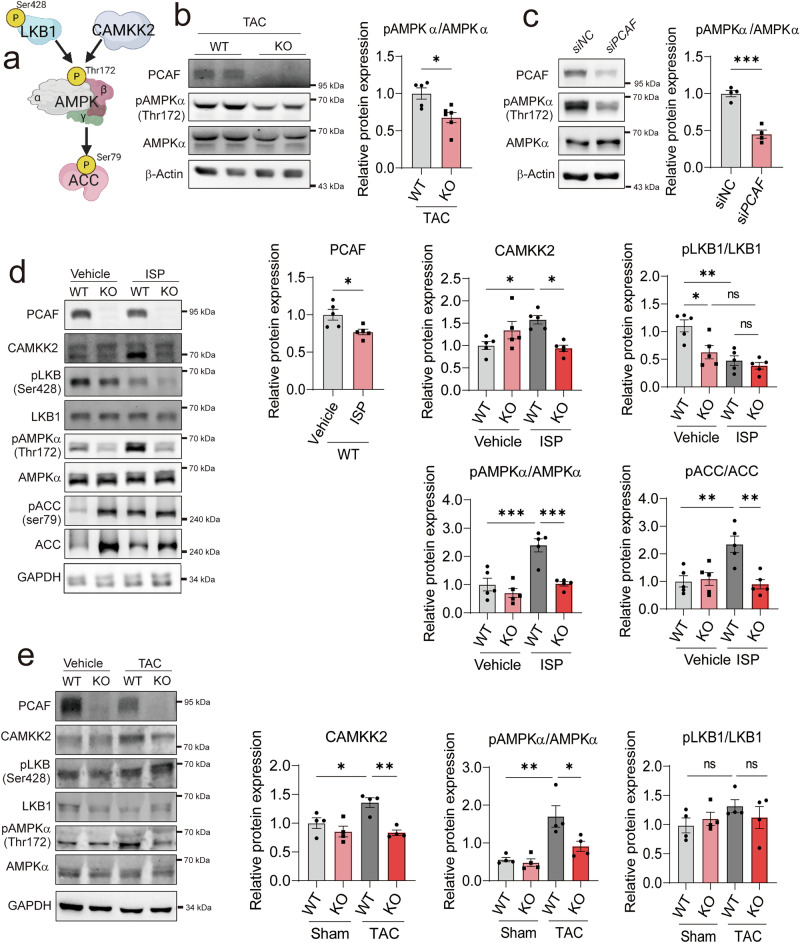
PCAF is involved in the AMPK signaling pathway. **a** A simplified scheme representing the AMPK signaling pathway. **b** Representative WB images in isolated cardiomyocytes and quantification of phosphorylated AMPKα (pAMPKα) at Thr172 from TAC- or sham-operated hearts (*n* = 5–6 per group). **c** Representative images of WB and quantification of pAMPKα at Thr172 in AC16 cells (*n* = 4 per group). **d** Representative WB images showing the AMPK signaling pathway in mouse hearts treated with vehicle or ISP, and quantification of PCAF, CAMKK2, phosphorylated AMPK α (Thr172), phosphorylated ACC (Ser79) and phosphorylated LKB1 (Ser428) (*n* = 5 per group). *P* values were determined using Student’s *t*-test for **c** and **e** and Tukey’s HSD test following one-way ANOVA for **d**. All data are presented as mean ± s.e.m. **e** Representative WB images showing the AMPK signaling pathway in mouse hearts subjected to sham or TAC surgery, and quantification of PCAF, CAMKK2, phosphorylated AMPKα (Thr172) and phosphorylated LKB1 (Ser428) (*n* = 4 per group). *P* values were determined using Tukey’s HSD test following one-way ANOVA. All data are presented as mean ± s.e.m.

Because the AMPK signaling pathway has been reported to prevent pathological hypertrophy by regulating energy homeostasis^7,9–11^, we checked alterations in the AMPK signaling pathway, as shown in Fig. 5a (ref. ^8,45^). We confirmed how AMPK was regulated in the PCAF-deficient mice. WB results showed that TAC induced a reduction in phosphorylation of AMPKα in PCAF-KO mice (Fig. 5b). In addition, PCAF downregulation in human cardiomyocyte AC16 cells resulted in a decrease in phosphorylation of AMPKα (Fig. 5c). Next, to investigate how PCAF regulates the phosphorylation of AMPK, we investigated the upstream kinases of AMPK.

Because PCAF functions as an acetyltransferase that modifies histones and other target proteins, we first examined whether AMPK itself is acetylated. Heart lysates from WT and KO mice were immunoprecipitated with an anti-acetyl lysine antibody to assess AMPK acetylation. As shown in Supplementary Fig. 7, AMPK acetylation was undetectable in both WT and KO hearts. Therefore, we considered that the regulation of AMPK phosphorylation by PCAF might occur indirectly through upstream kinases.

Recent studies indicate a role for adrenergic receptors in regulating AMPK activity, suggesting that adrenergic signaling may be a critical modulator of metabolic and energy homeostasis pathways^46–48^. Hence, we aimed to investigate the detailed signaling pathways in ISP-treated mice. WB results revealed that ISP treatment significantly reduced the expression of PCAF and AMPKα, but elevated phosphorylation of AMPKα at Thr172, indicative of AMPKα activation. Consistently, phosphorylation of ACC, a downstream target of AMPKα, was increased by ISP in WT mice, while phosphorylation of ACC disappeared in PCAF-KO mice (Fig. 5d). So far, two different kinases, LKB1 and CAMKK2, have been identified as well-known upstream regulators of AMPKα (Fig. 5a). To confirm the responsible upstream kinases for suppressed phosphorylation of AMPKα in PCAF-KO mice, we examined the change in LKB1 and CAMKK2 expression. Intriguingly, CAMKK2 expression in WT mice was significantly augmented by ISP treatment, while ISP-induced increase in CAMKK2 expression was blocked in PCAF-KO mice. However, phosphorylation of LKB1 at Ser428, an active marker of LKB1, was markedly inhibited in ISP-treated WT mice, which was not correlated with AMPKα phosphorylation (Fig. 5d).

We next examined whether similar signaling alterations occurred under pressure-overload-induced cardiac hypertrophy. As shown in Fig. 5e, TAC stimulation also activated a comparable signaling cascade. The protein level of CAMKK2 was increased by TAC in WT mice, whereas this increase was abolished in PCAF-deficient hearts (Fig. 5e, left bar graph). Likewise, phosphorylation of AMPK, a downstream effector of CAMKK2 signaling, was elevated by TAC in WT hearts but was not observed in PCAF-KO mice (middle bar graph). By contrast, although phosphorylation of LKB1 was decreased by ISP treatment and remained unchanged by PCAF deficiency (right bar graph), TAC did not affect LKB1 phosphorylation, nor was it influenced by PCAF deletion. In short, these data indicate that CAMKK2, rather than LKB1, is responsible for regulation of AMPKα activity under cardiac stresses.

### PCAF acetylates and activates CAMKK2, thereby promoting calcium-mediated activation of CAMKK2

**Fig. 6:**
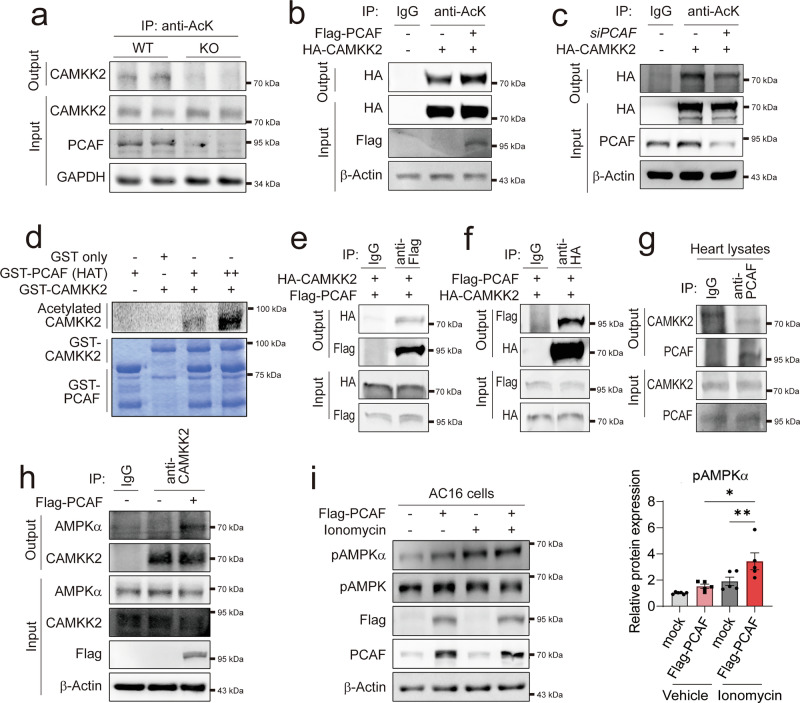
PCAF-mediated acetylation of CAMKK2 is essential for its activation. **a** IP-based acetylation assay showing the acetylation level of CAMKK2 in cardiomyocytes isolated from WT or PCAF-KO mice. **b** Confirmation of acetylation changes in HA-CAMKK2 upon PCAF overexpression in HEK293T cells. **c** Confirmation of acetylation changes in HA-CAMKK2 following PCAF knockdown in HEK293T cells. **d** In vitro acetylation assay showing acetylation of GST-CAMKK2 by GST-PCAF (HAT domain). **e** Co-IP analysis showing the interaction between Flag-PCAF and HA-CAMKK2 in HEK293T cells. **f** Reciprocal co-IP analysis confirming the interaction between HA-CAMKK2 and Flag-PCAF in HEK293T cells. **g** Co-IP showing the endogenous interaction between PCAF and CAKK2 in the mouse heart. **h** IP assay showing the interaction between CAMKK2 and AMPKα upon PCAF overexpression in AC16 cells. **i** WB analysis of AC16 cells transfected with Flag-PCAF and treated with either vehicle or ionomycin (Iono) (*n* = 5–6 per group), with quantification of phosphorylated AMPKα (pAMPKα). *P* values were determined using Tukey’s HSD test following one-way ANOVA. All data are presented as mean ± s.e.m.

To confirm the cellular distribution of PCAF, we first examined its subcellular localization in mouse hearts. PCAF was found in both the nucleus and the cytosol but was absent from the mitochondria (Supplementary Fig. 8a). To determine whether PCAF regulates the expression of CAMKK2 through transcriptional regulation, we measured the mRNA levels of CAMKK2. There was no significant difference in CAMKK2 mRNA levels between WT and PCAF-KO mice under either ISP or TAC conditions (Supplementary Fig. 8b,c). Subsequently, we assessed the acetylation status of CAMKK2 to explore whether PCAF modulates CAMKK2 via its acetyltransferase activity. Intriguingly, a reduction in acetylation of CAMKK2 was observed in PCAF-KO mice (Fig. 6a). Thus, we checked whether PCAF can acetylate CAMKK2 and whether acetylation of CAMKK2 can affect AMPK activity. Indeed, PCAF induced acetylation of CAMKK2 in a dose-dependent manner (Fig. 6b), and PCAF knockdown abolished acetylation of CAMKK2 (Fig. 6c). In vitro acetylation assay demonstrated that PCAF directly acetylated CAMKK2 (Fig. 6d). Taken together, these data suggest that CAMKK2 is an acetylation target of PCAF. Next, we confirmed whether PCAF directly interacts with CAMKK2. An IP assay demonstrated that PCAF associates with CAMKK2 in vitro (Fig. 6e,f) and in the heart (Fig. 6g). Then, we postulated that PCAF-mediated acetylation of CAMKK2 may affect the interaction of CAMKK2 with AMPK. IP analysis showed that PCAF overexpression enhanced the interaction of CAMKK2 and AMPKα at the basal level (Fig. 6h). Then, to check whether PCAF-mediated acetylation of CAMKK2 affects its activity, human cardiomyocyte AC16 cells were transfected with PCAF, followed by treatment with ionomycin, which activates CAMKK2 through Ca^2+^ influx^8^. We confirmed that PCAF overexpression per se induced AMPKα phosphorylation at Thr172, which was enhanced by ionomycin (Fig. 6i, top), and the corresponding quantification data are shown in the bar graph in Fig. 6i. Collectively, these data suggest that PCAF-mediated acetylation is essential for calcium-dependent activation of CAMKK2.

### Pharmacological activation of PCAF protects hearts from TAC-induced pathological cardiac remodeling

**Fig. 7:**
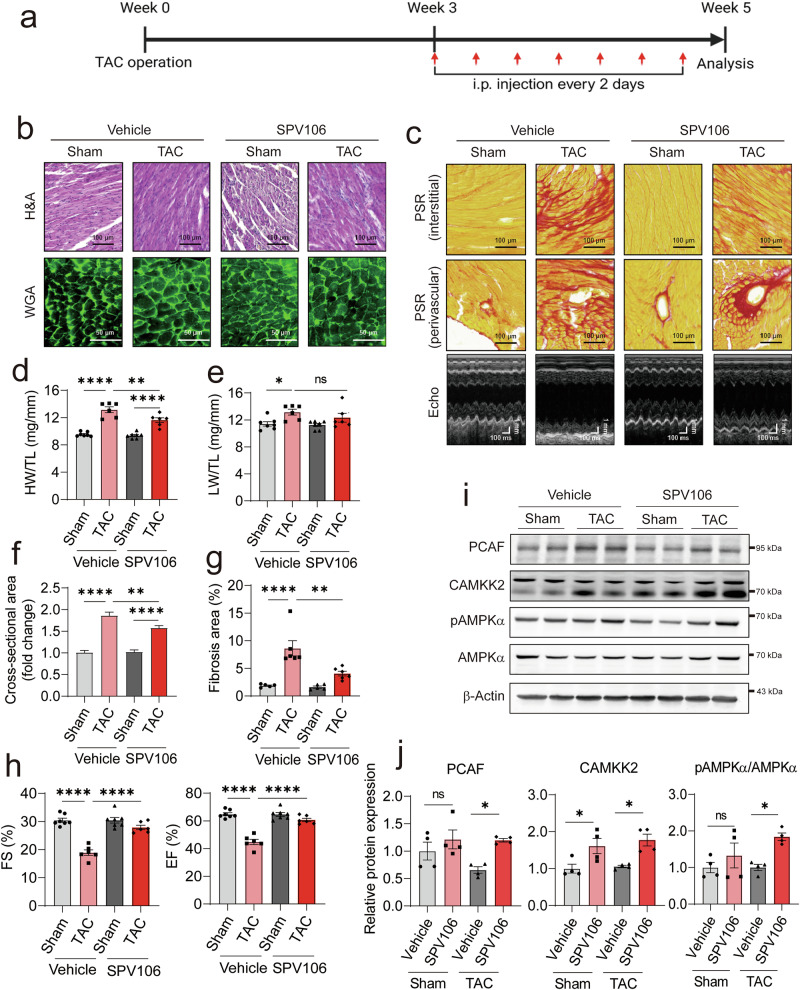
Pharmacological activation of PCAF ameliorates TAC-induced cardiac hypertrophy and dysfunction. **a** Experimental design schematic. After surgery, mice were received intraperitoneal injection of SPV106 (20 mg/kg) every 2 days. **b** Representative histological images (H&E and WGA staining). **c** Representative images of PSR staining and echocardiography results. **d**, **e** Quantification of HW/TL (**d**) and LW/TL (**e**) (*n* = 6–7 per group). **f** Cross-sectional area of cardiomyocytes analyzed by WGA staining (*n* = 63–100 per group). **g** Fibrosis area (*n* = 5–6 per group). **h** Echocardiographic analysis of FS and EF (*n* = 6–7 per group). *P* values in were determined using Tukey’s HSD test following one-way ANOVA. All data are presented as mean ± s.e.m. **i** Representative WB images showing the AMPK signaling pathway in mouse hearts with vehicle or SPV106. **j** Quantification of PCAF, CAMKK2 and phosphorylated AMPKα (pAMPKα, Thr172) expression (*n* = 4 per group).

Because our findings suggested that PCAF acts as a key molecule of cardiac remodeling, we tested whether the selective activation of PCAF prevents cardiac hypertrophy and dysfunction. After 3 weeks of TAC surgery, mice were intraperitoneally injected with vehicle or SPV106, a selective PCAF activator^49^, every 2 days for 2 weeks, as illustrated in Fig. 7a. After TAC surgery, administration of SPV106 to mice caused significant attenuation of hypertrophy and fibrosis (Fig. 7b,c). TAC-induced increases in HW/TL and LW/TL were attenuated by administration of SPV106 (Fig. 7d,e). TAC-induced increases in the cross-sectional area of cardiomyocytes (Fig. 7f) and fibrosis area were reduced by SPV106 (Fig. 7g). TAC-induced cardiac contractility dysfunction was improved by SPV106 administration, as measured by FS and EF (Fig. 7h).

In Figs. 5 and 6, we demonstrated that PCAF functions upstream of CAMKK2 and AMPK, activating CAMKK2 and thereby promoting AMPK phosphorylation. To determine how this downstream signaling pathway is affected by SPV106, we examined CAMKK2 and AMPK in hearts treated with SPV106 after TAC surgery. As shown in Fig. 7i, the amount of CAMKK2 was increased by SPV106 alone (lanes 1 and 2 versus lanes 5 and 6 in the second gel) and was further elevated by TAC (lanes 5 and 6 versus lanes 7 and 8). Phosphorylation of AMPK was also enhanced by SPV106 (see third and fourth gels in Fig. 7i). The corresponding quantification data are shown in Fig. 7j. These data imply that pharmacological activation of PCAF by SPV106 rescues the progression of cardiac hypertrophy and dysfunction induced by TAC surgery.

## Discussion

This study provides new insights into how PCAF regulates cardiac hypertrophy and heart failure through direct modulation of CAMKK2 via acetylation. Analysis of the GEO database revealed that PCAF expression is significantly reduced in patients with DCM. This finding suggests a potential link between PCAF downregulation and the pathological mechanisms of DCM, implying that PCAF may play a crucial role in maintaining cardiac homeostasis and its dysregulation could contribute to disease progression. In the progression of TAC-induced pathological cardiac remodeling, PCAF expression was markedly reduced, indicating the linkage of its dysregulation to adverse structural changes and compromised myocardial function. These findings highlight PCAF as a molecular checkpoint against pathological remodeling, and its presence appears to be critical for maintaining cardiac integrity under pathological stress. Our study demonstrates that PCAF deficiency exacerbates cardiac remodeling and impairs cardiac contractility by inducing left ventricular wall thinning, which suggests the development of eccentric hypertrophy. Upon hypertrophic stress, PCAF-KO mice showed significant increases in heart and lung weights and reduced cardiac function, suggesting that the loss of PCAF led to rapid progression of HFrEF. These results collectively underscore the cardioprotective role of PCAF and emphasize its role as a regulator of maladaptive cardiac remodeling.

Surprisingly, our findings demonstrate that PCAF-mediated acetylation of CAMKK2 is essential for its activation to regulate AMPK, a key metabolic regulator that protects against pathological hypertrophy by maintaining cellular energy homeostasis^7–11^. In PCAF-KO mice, we observed a reduction in CAMKK2 acetylation, which subsequently resulted in diminished AMPK activation. This downregulation of the AMPK signaling cascade in the absence of PCAF suggests that CAMKK2 acetylation is a critical step in maintaining AMPK’s protective effects against cardiac stress. These results establish a novel mechanism in which PCAF modulates cardiac metabolism and highlights PCAF as an essential modulator of cardiac hypertrophy.

Our findings suggest that CAMKK2 can be acetylated and functionally regulated by PCAF. It is well established that CAMKK2 is tightly regulated by intracellular Ca^2+^ and calmodulin, a calcium-sensing protein^50^. Although this study did not investigate the effects of PCAF on calcium levels or its direct or indirect effect on calcium signaling, it is plausible that the observed reduction in contractile function in PCAF-KO mice may involve dysregulated calcium handling and signaling mediated by the PCAF–CAMKK2 axis. Therefore, further studies are needed to elucidate this potential mechanism.

Our data suggest that PCAF interacts with CAMKK2, potentially through specific regions of the protein. Although we did not conduct experiments using truncated mutants to identify the domain responsible for this interaction, the results indicate that this interaction may involve distinct structural or sequence features. This raises questions about the structural requirements and post-translational modifications that enable PCAF to recognize and selectively acetylate CAMKK2. However, the specific lysine residues on CAMKK2 that undergo acetylation and their effects on CAMKK2 activation remain to be identified. In addition, a study on CAMKK2 activity-dead mutant mice revealed exacerbated cardiac hypertrophy after TAC^12^, suggesting that CAMKK2 activity plays a protective role against cardiac hypertrophy. Because the acetylation of CAMKK2 is still largely uncharacterized, further research is needed to clarify its regulatory role within the AMPK pathway.

In our previous study, we demonstrated that PCAF acetylates HDAC2 in response to hypertrophic stimuli in primary neonatal cardiomyocytes^15^, thereby activating HDAC2 and promoting cardiac hypertrophy. At first glance, these results appear to contradict our current findings, in which PCAF KO unexpectedly exacerbated cardiac remodeling and led to abrupt HFrEF. However, a more careful interpretation suggests that these findings may, in fact, reflect distinct aspects of PCAF’s physiological function. In many tissues, activation of cell survival signaling pathways leads to enhanced cell proliferation, whereas in terminally differentiated cardiomyocytes, similar signaling instead manifests as a hypertrophic phenotype^51–54^. The PCAF-induced hypertrophy observed in our earlier study probably represents such an adaptive, prosurvival response. By contrast, in the absence of PCAF, as in the PCAF KO-mice of the present study, this protective mechanism appears to be lost, leading to rapid progression toward pathological remodeling characterized by ventricular dilation, wall thinning and systolic dysfunction, features typical of HFrEF. Taken together, these findings suggest that PCAF may function as a molecular switch governing the transition from adaptive cardiac hypertrophy to overt heart failure.

In addition to this hypothesis, several other factors may contribute to the apparent differences between our previous in vitro results and the current in vivo findings. These include the distinct properties of neonatal versus adult cardiomyocytes, differences in gene regulation between transient transfection and genetic deletion models, postnatal upregulation of PCAF expression, and the potential compensatory role of GCN5, which has been reported to exacerbate cardiac hypertrophy and dysfunction^55–61^. Further studies are warranted to delineate these mechanisms and to clarify the precise role of PCAF in maintaining cardiac homeostasis.

Although we suggested the effect of CAMKK2 acetylation by PCAF in AMPK activation, further research is needed to clarify how PCAF–CAMKK2 interactions impact cardiac energy metabolism under various stress conditions, including chronic ischemia and metabolic syndromes. Our findings suggest that the PCAF activator SPV106 can mitigate hypertrophy, highlighting its potential therapeutic benefits. In-depth structural and biochemical studies could further clarify the precise binding motifs on CAMKK2 that facilitate its interaction with PCAF and reveal how these interactions respond to cellular stress signals. In addition, investigating whether the PCAF–CAMKK2–AMPK axis functions in other cell types involved in cardiac remodeling, such as cardiac fibroblasts, may provide broader insights into cardioprotective mechanisms.

In the present study, the cardiomyocyte-specific PCAF KO as well as global KO models revealed that loss of PCAF accelerates maladaptive remodeling and precipitates HFrEF. Building on these findings, further in vivo studies using temporally controlled or tissue-specific inducible models will help to define the timing and systemic aspects of PCAF–CAMKK2 interactions during disease progression. Furthermore, if PCAF indeed acts as a molecular switch controlling the transition from adaptive cardiac hypertrophy to HFrEF, it may provide a new mechanistic insight into the pathogenesis of cardiac hypertrophy or heart failure with preserved EF, where systolic function is relatively well preserved. This perspective suggests that the loss of PCAF function accelerates maladaptive remodeling characterized by ventricular dilation and wall thinning. Therefore, restoring PCAF activity, either pharmacologically through agents such as SPV106 or by mimicking CAMKK2 acetylation, could potentially delay the onset of left ventricular dilatation and progression to HFrEF. Ongoing studies in our laboratory aim to validate these hypotheses and explore whether modulating PCAF function could offer new therapeutic avenues for both metabolic and structural preservation in heart disease.

## Supplementary information

Supplementary Information
