# Supplementary material for: Loss of p300/CBP-associated factor aggravates cardiac remodeling via regulation of CAMKK2 acetylation
Source: Exp Mol Med. 2026 Apr 20;58(4):1297–310. doi: 10.1038/s12276-026-01698-z (PMC13144538; doi:10.1038/s12276-026-01698-z)
Supplement: Supplementary file 1 — Supplementary Information [file 12276_2026_1698_MOESM1_ESM.pdf]

Supplementary Methods

TAC surgery and echocardiography

For TAC surgery, the mice were anaesthetized with isoflurane (1.0–1.5% inhalation) and then intubation was carried out. A longitudinal skin incision was made, followed by removal of the sternum to locate the thymus and aorta. The thymus was retracted, and then the arch of aorta between brachiocephalic and left common carotid artery tied by a 7-0 silk suture with 27-gauge needle. Consequently, the needle was immediately removed and the incision was sutured using a 4-0 silk sutures. As a control, sham mice were subjected to same operation without ligation.

In the current study, mice were temporarily anesthetized by Avertin and cardiac function was measured by two-dimensional transthoracic echocardiography (XCUBE60, ALPINION, Seoul, South Korea). Echocardiographic analysis was conducted 4 weeks after TAC surgery using an XCUBE60 echocardiography system (ALPINION) equipped with an L10-25H linear transducer (ALPINION). To minimize potential bias, all measurements were performed by an experienced investigator blinded to the experimental conditions. Two-dimensional guided M-mode images of the left ventricle (LV) were acquired from the parasternal view. LV internal dimensions, as well as the thickness of the LV free wall and interventricular septum, were measured. The percentage of LV fractional shortening (LV%FS) was calculated using the formula: LV%FS = [(LVDd − LVDs)/LVDd] × 100, where LVDd and LVDs represent LV dimensions at end-diastole and end-systole, respectively. The LV ejection fraction (LV%EF) was determined as: LV%EF = [(EDV − ESV)/EDV] × 100, where EDV and ESV indicate LV

volumes at end-diastole and end-systole. LV volumes were estimated according to the Teicholz method. To harvest tissue, the mice were sacrificed by carbon dioxide inhalation.

Isolation of mouse cardiac fibroblasts and myocytes

The mice were anesthetized, and hearts were collected. The heart was immediately washed with Ca^{2+}-free Tyrode buffer (137 mmol NaCl, 5.4 mmol KCl, 10 mmol HEPES pH 7.4, 1 mmol $MgCl_2$, 5 mmol taurine, 10 mmol glucose, and 10 mmol BDM) for 5 minutes, followed by perfusion with enzyme solution (0.35 U/mL collagenase type B and 0.1 mg/mL hyaluronidase in Ca^{2+}-free Tyrode buffer) for an additional 20 minutes. After perfusion, the heart tissue was dissociated, and resuspended in 5% BSA in Tyrode buffer for 10 minutes, then passed through a 100-µm cell strainer (#352360, Falcon, NY, USA). The suspension was sedimented by gravity for 10 minutes to enrich cardiomyocytes. The supernatants were transferred to a new tube to collect purified myocytes.

Cell culture and transfection

HEK293T and H9c2 cells were purchased from the American Type Culture Collection (ATCC, Manassas, VA, USA). AC16 cells were purchased from Sigma. Cells were cultured in Dulbecco's modified Eagle's medium (DMEM, #LM001-05, Welgene, Gyeongsan, Korea) supplemented with 10% fetal bovine serum (FBS, #SH30919.03, Hyclone, Waltham, MA, USA) and 1% antibiotics (penicillin and streptomycin) (#15240-0662, Gibco, Thermo Fisher Scientific, Waltham, MA, USA) at 37°C in a humidified incubator with 5% CO2. Subculture was performed by trypsinization with TrypLE™ Express (#12605, Gibco). For treatment, cells were starved, followed by treatment with ionomycin (#I24222, Invitrogen, Waltham, MA, USA).

The PCAF vector (pCI-Flag-PCAF) was purchased from Addgene (#8941, Watertown, MA, USA). The overexpression vector of CAMKK2 (pPM-C-HA-CAMKK2) was from Applied Biological Materials (Abm, Richmond, BC, Canada). Cells were transfected using Lipofectamine 3000 (#L3000001, Thermo) according to the manufacturer's instruction.

Small interfering RNAs (siRNAs) against rat *Pcaf*, predesigned siRNA for human *PCAF* (#8805) and AccuTarget negative control siRNA (#SN-1003) were obtained from Bioneer (Daejeon, Korea). Transfection of siRNA was carried out using Lipofectamine RNAiMax Transfection Reagent (#13778075, Thermo) according to the manufacturer's protocol.

Histology, immunohistochemistry (IHC)

Mouse hearts were fixed in 4% paraformaldehyde at 4°C for 24 hours, and then were dehydrated through a series of ethanol baths, followed by paraffin embedding. The hearts were cross-sectioned and stained with hematoxylin and eosin (H&E) and Picrosirius red (PSR) staining (#ab150681, Abcam, Waltham, MA, USA), according to the manufacturer's instruction. The fibrotic area was determined using Image J software. For wheat germ agglutinin (WGA) staining, paraffin-embedded heart tissues were prepared and stained with WGA (#11261, sigma) to visualize the plasma membrane of cardiomyocytes. Staining images were acquired with an Axio Scan.Z1 scanner (Carl Zeiss Microscopy, GmbH, Jena, Germany) and a laser scanning microscope (DE/ LSM700, Carl Zeiss Microscopy).

RNA extraction and quantitative real-time PCR

Total RNA was prepared using the TRI reagent (#TR118, Molecular Research Center, OH, USA), according to the manufacturer's instruction. Extracted RNA was incubated in DNase I

(#2270A, Takara, Tokyo, Japan) to remove residual DNA. A complementary DNA was obtained using RevertAid reverse transcriptase (#EP0442, Thermo) and random hexamers (#SO142, Thermo). qRT-PCR was performed by using the QuantiTect SYBR Green PCR kit (#204143, Qiagen, Germantown, MD, USA) with specific primers and a Rotor-Gene Q real-time PCR cycler (Qiagen). The specific primers were purchased from Bioneer. The used primers are listed in Supplementary Table 1.

WB analysis and immunoprecipitation (IP)

Radioimmunoprecipitation (RIPA) buffer (#R2002, Biosesang, Seongnam, Korea) was used for cell lysis. The Pierce bicinchoninic acid protein assay kit (#23225, Thermo) was used to determine protein concentration in lysates. For the WB assay, protein samples were prepared by mixing lysate with 4× NuPAGE SDS sample buffer (#LC2676, Invitrogen), followed by boiling at 95 ℃ for 5 minutes. The protein samples were separated by SDS-polyacrylamide gel, followed by transfer to methanol-activated PVDF membranes (#IPVH00010, Merck). The membranes were incubated with the indicated primary antibody at 4 ℃, and further incubated with horseradish peroxidase-conjugated secondary antibody. To detect the protein of interest, immobilon chemiluminescent HRP substrate (#P90720, Merck) and FUSION-FX-SPECTRA (Vilber GmbH, Eberhardzell, Germany) were used. Band intensities were calculated using Image J software.

For the IP assay, the lysate was precleared by protein A/G PLUS-Agarose (sc2003, Santa), then briefly centrifuged to remove beads. The precleared lysates were incubated with the primary antibody with gentle shaking at 4 ℃. To enrich the antibody-protein complex,

Protein A/G PLUS-Agarose (#sc-2003, Santa) was added and further incubated for 2 hours. The bead complex was centrifuged and washed with the lysis buffer. The immobilized immune complexes were mixed with SDS sample buffer (#LC2676, Invitrogen) with β-mercaptoethanol and subjected to boiling. The corresponding purified IgG was used as a negative control.

Supplementary Figures

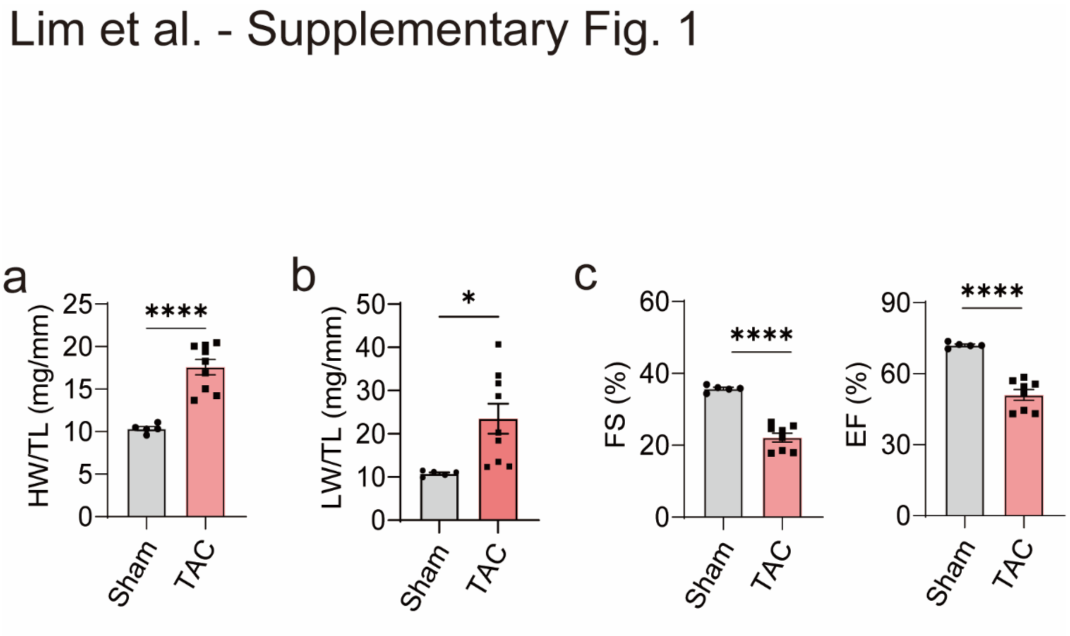

Supplementary Fig. 1. Cardiac hypertrophy and dysfunction in mice after TAC surgery. (a) The Heart weight-to-tibia length ratio (HW/TL) was significantly increased in mice subjected to transverse aortic constriction (TAC) compared with sham-operated controls, indicating cardiac hypertrophy. (b) The Lung weight to tibia length ratio (LW/TL) was elevated in TAC mice, suggesting pulmonary congestion secondary to heart failure. (c) Echocardiographic analysis revealed markedly decreased fractional shortening (FS) and ejection fraction (EF) in TAC mice compared with sham controls, confirming impaired systolic function. *p*–values were determined using Tukey's HSD test following one -way ANOVA. Data are presented as mean ± SEM.

Lim et al. - Supplementary Fig. 2

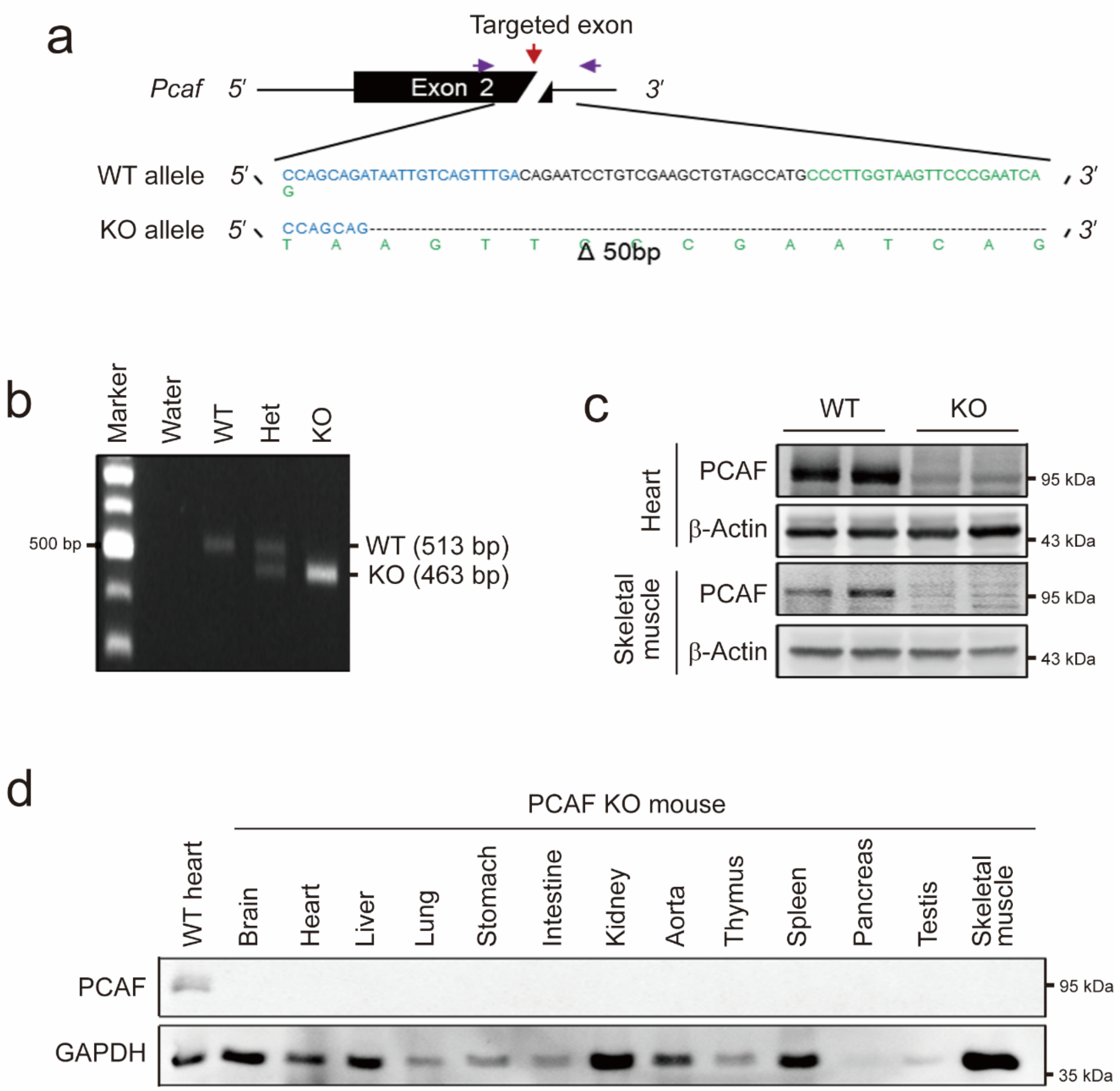

Supplementary Fig. 2. Strategy for global deletion of *Pcaf* in mice. (a) The schematic illustration showing the targeting strategy used to generate the *Pcaf* knockout (KO) mouse. (b) Conventional PCR-based genotyping of PCAF KO mice. WT mice exhibited a single band of 513 bp, PCAF KO mice showed a single band of 463 bp. Het indicates heterozygous mice. (c) WB analysis showing PCAF expression in hearts and skeletal muscles of WT and KO mice. (d) WB analysis showing PCAF expression in various organs of the PCAF KO

mice.

Lim et al. Supplementary Fig. 3

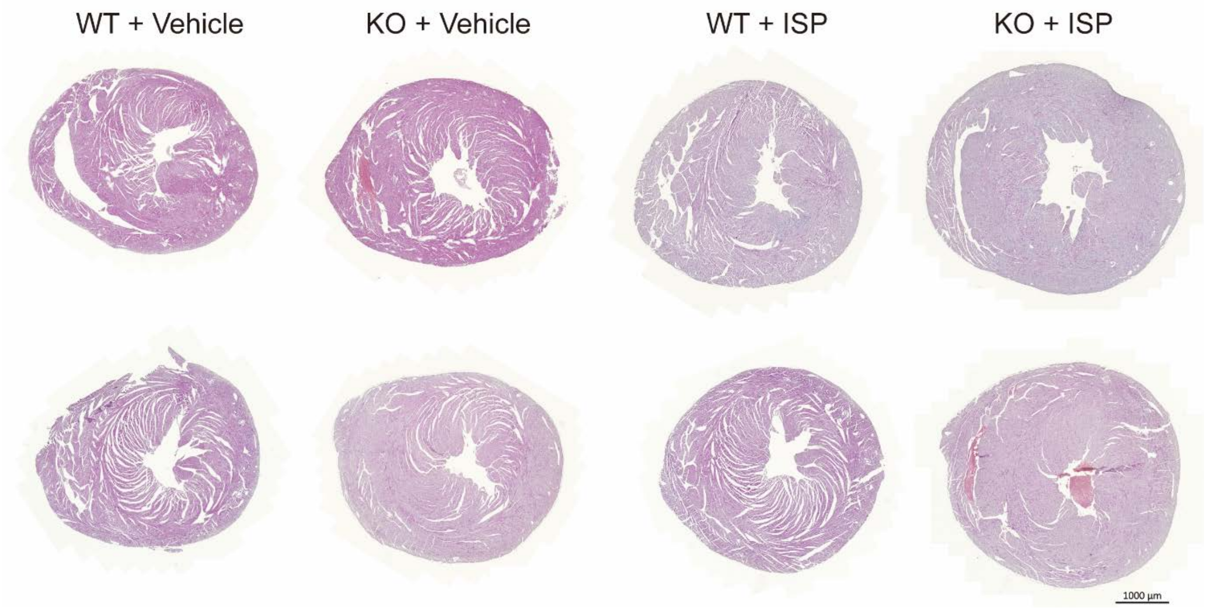

Supplementary Fig. 3. Chronic ISP infusion induces cardiac hypertrophy in WT mice, which is further exacerbated in global PCAF KO mice. Representative H&E–stained transverse heart sections from WT and global PCAF KO mice after 6 days of isoproterenol (ISP, 30 mg/kg/day) infusion via osmotic pumps. ISP treatment induced marked cardiac hypertrophy in WT mice compared with vehicle-treated controls, and this response was further exaggerated in PCAF-deficient hearts, showing enhanced hypertrophy. Scale bar, 1000 μm

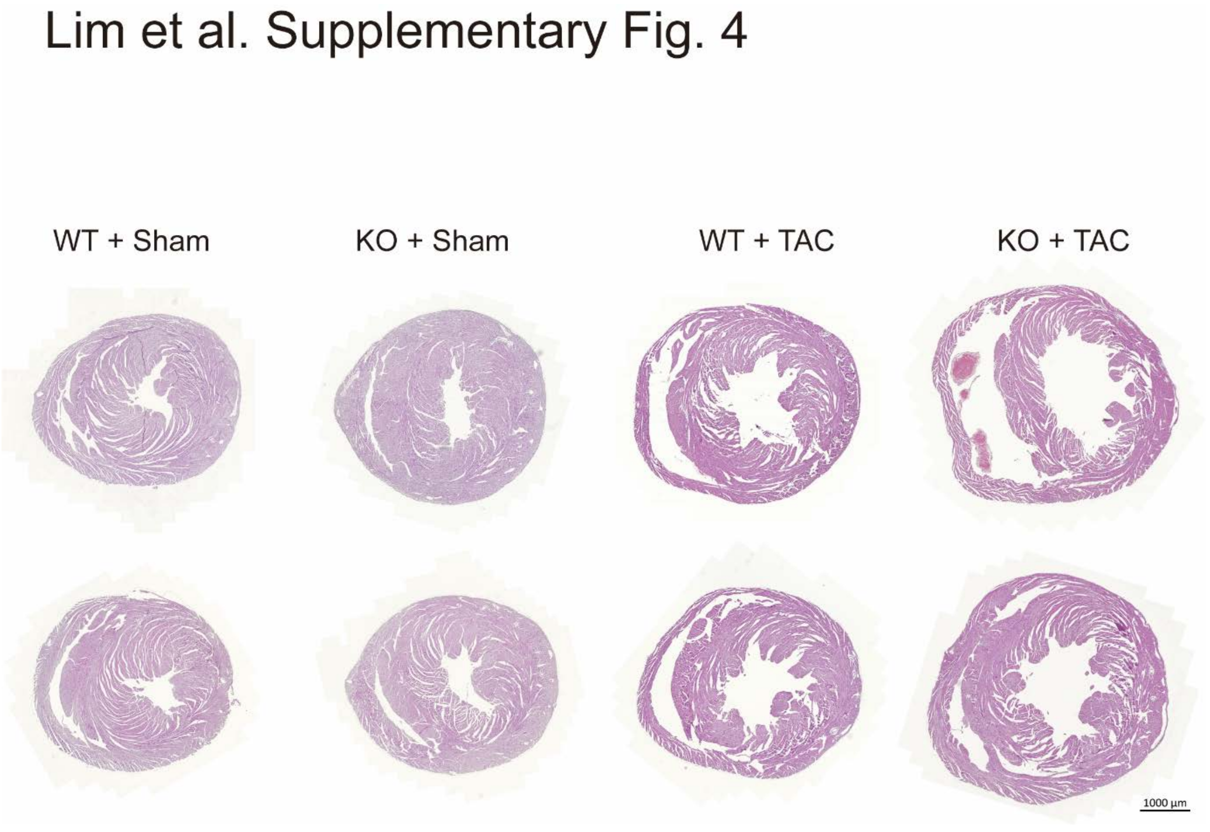

Supplementary Fig. 4. TAC surgery induces concentric cardiac hypertrophy in WT mice, whereas PCAF deficiency leads to exaggerated eccentric hypertrophy with wall thinning. Representative H&E-stained heart sections from WT and PCAF global KO mice after sham or TAC surgery. TAC induced marked concentric hypertrophy in WT hearts, while PCAF-deficient hearts exhibited an exaggerated eccentric hypertrophic phenotype characterized by ventricular dilation and wall thinning. Scale bar, 1000 μm

Lim et al. - Supplementary Fig. 5

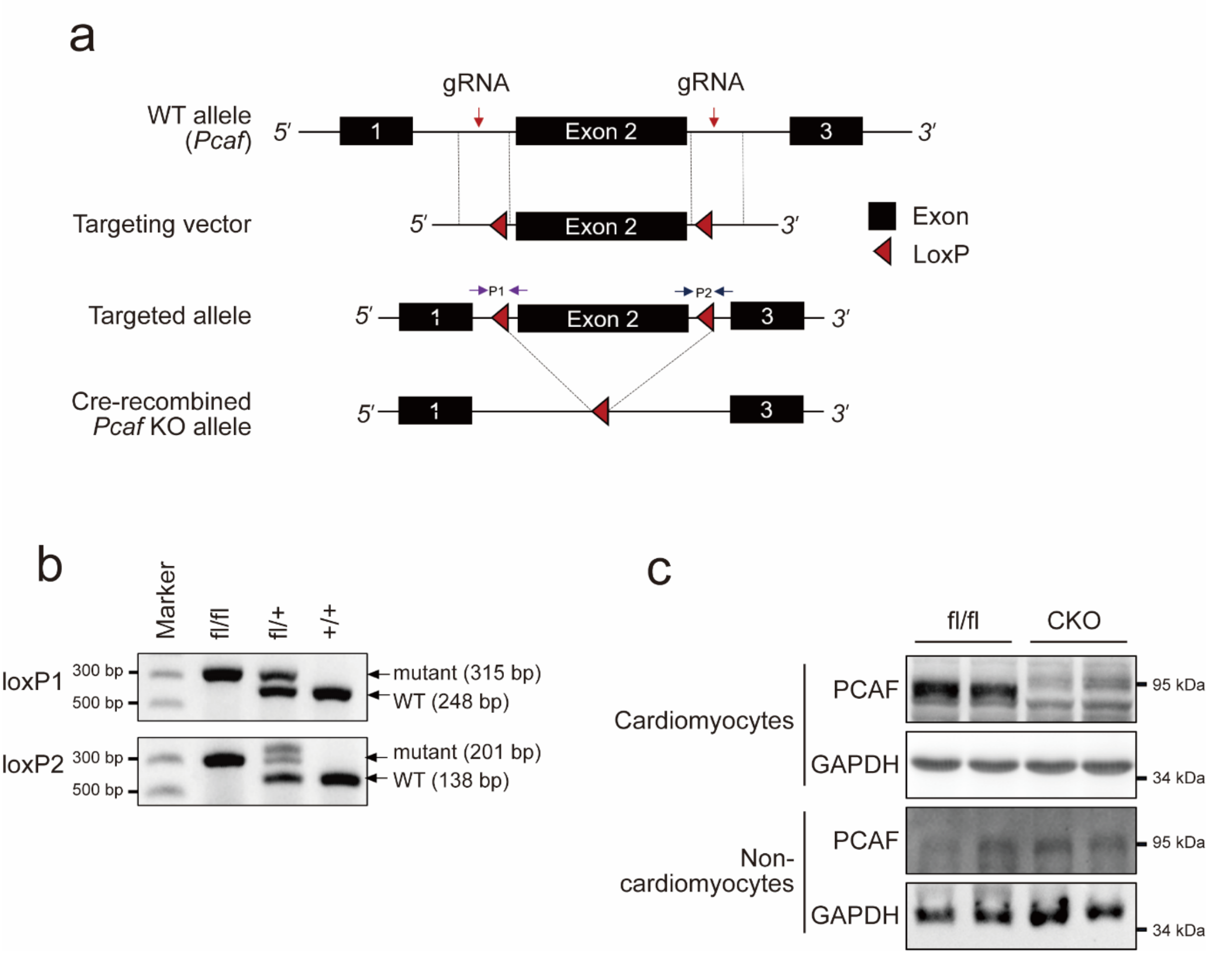

Supplementary Fig. 5. The strategy for geniting cardiomyocyte-specific *Pcaf* knockout mice. (a) Schematic illustration of the targeting strategy used to generate the *Pcaf* floxed allele. Two gRNAs were designed to flank exon 2 of the *Pcaf* gene, and *loxP* sites were inserted flanking this exon using a targeting vector. Cre-mediated recombination deleted exon 2, resulting in the cardiomyocyte-specific *Pcaf* knockout allele. (b) Genotyping analysis of WT (+/+), heterozygous floxed (fl/+), and homozygous floxed (fl/fl) mice. PCR amplification using primer pairs P1 and P2 produced bands of 248 and 138 bp for the WT allele, and 315 and 201 bp for the mutant (floxed) allele, respectively. (c) WB analysis showing PCAF protein expression in isolated cardiomyocytes and non-cardiomyocytes from fl/fl and CKO mouse. PCAF expression was markedly reduced in CKO cardiomyocytes, confirming

successful cardiomyocyte-specific deletion of *Pcaf*.

Lim et al. Supplementary Fig. 6

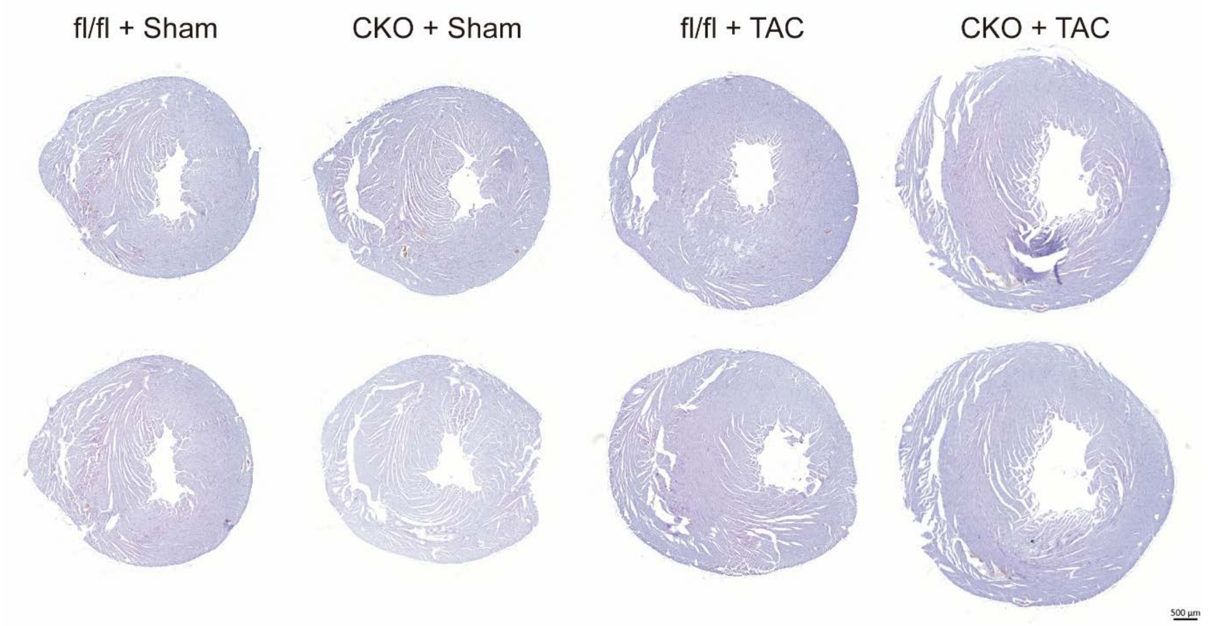

Supplementary Fig. 6. Histological assessment of cardiac morphology in f/f and CKO mice following TAC. Representative H&E–stained transverse heart sections from f/f and CKO mice subjected to sham or TAC surgery. Both f/f and CKO hearts showed normal morphology under sham conditions. After TAC, f/f mice exhibited concentric cardiac hypertrophy with thickened ventricular walls, which was further exaggerated in CKO mice. Scale bar, 500 µm.

Lim et al. - Supplementary Fig. 7

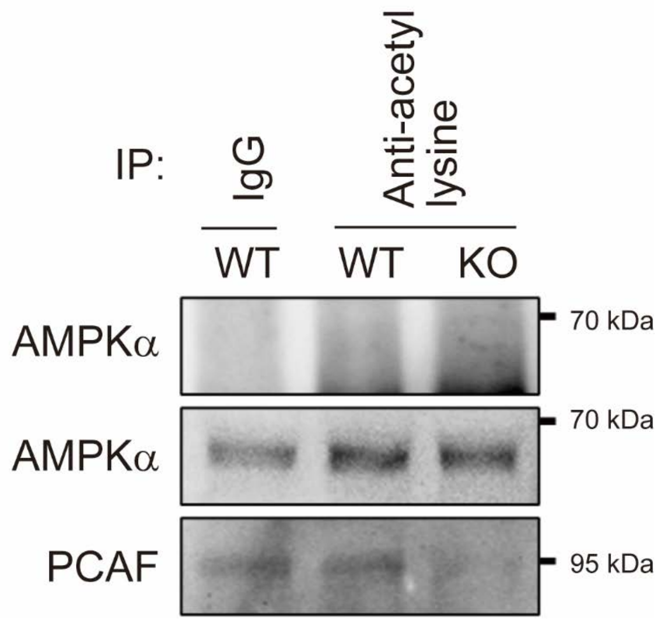

Supplementary Fig. 7. No detectable acetylation of AMPKα in the mouse heart. IP-based acetylation assay showing no detectable acetylation of AMPKα in the hearts of WT or PCAF KO mice.

Lim et al. - Supplementary Fig. 8

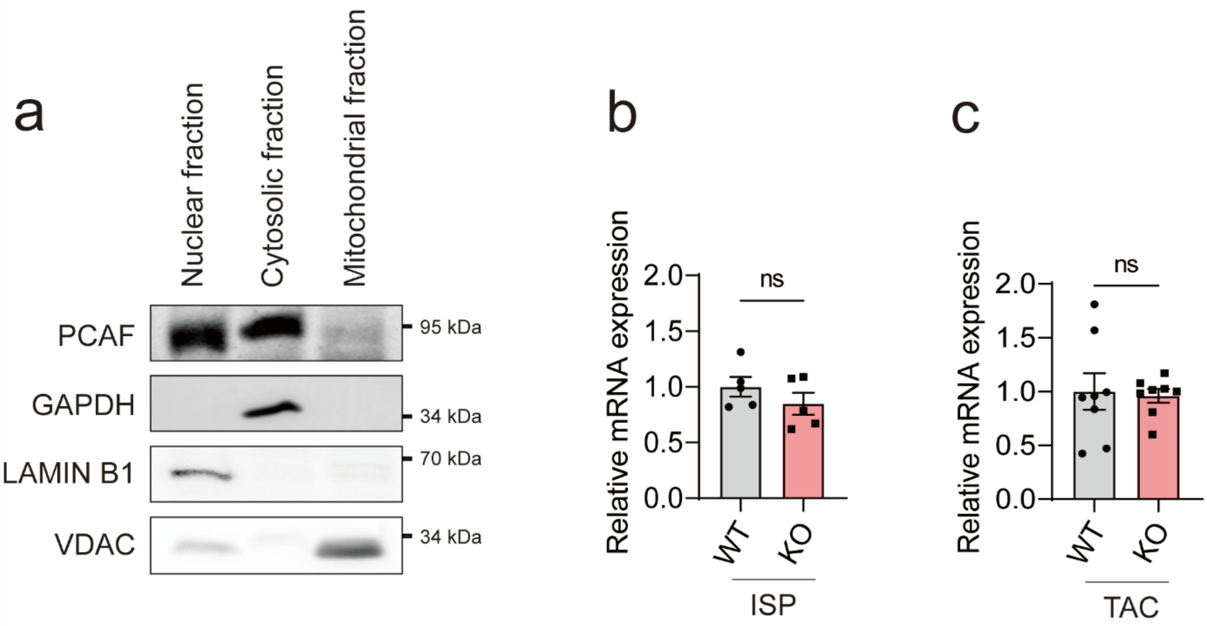

Supplementary Fig. 8. Subcellular localization of PCAF in mouse adult cardiomyocytes, and lack of effect of PCAF deletion on CAMKK2 mRNA expression after ISP or TAC treatment. (a) Subcellular fractionation of mouse heart tissue. GAPDH, LAMINB1, and VDAC were used as cytosolic, nuclear, and mitochondrial markers, respectively. (b-c) Relative CAMKK2 mRNA expression in the hearts of WT and PCAF KO mice after ISP infusion or TAC surgery (n = 5 per group). *p*–values were determined by using Tukey's HSD test following one-way ANOVA. Data are presented as mean ± SEM.

Supplementary Table

Supplementary Table 1. Real-time PCR primer sequence used in this study

Mouse

**Gene**	**Forward (5' to 3')**	**Reverse (5' to 3')**
Pcaf	GCCGTGTCATTGGTGGTATC	GGGTTCCATAGCCCTTGACT
Nppa	TGGAGCCCAGAGTGGACTAGG	AGCTGCGTGACACACCACAA
Nppb	TCTGTCACCGCTGGGAGGTC	TCTGGGCCATTTCCTCCGACT
Camkk2	TTGCTGTCTAACACCGTGG	GGCCTGACTCTTGATCTTACTG
Gapdh	GCATGGCCTTCCGTGTTCCT	CCCTGTTGCTGTAGCCGTATTCAT

**Genotyping**	**Forward (5' to 3')**	**Reverse (5' to 3')**
Pcaf	ACTTAGCTCACGTGTGAGTC	CCTCCACCAGGACAAATCAG
